# Chirality-modulated photonic spin Hall effect in PT-symmetry

**DOI:** 10.1515/nanoph-2022-0229

**Published:** 2022-06-28

**Authors:** Chengkang Liang, Dongxue Liu, Rao Liu, Dongmei Deng, Guanghui Wang

**Affiliations:** Guangdong Provincial Key Laboratory of Nanophotonic Functional Materials and Devices, South China Normal University, Guangzhou, China; Guangzhou Key Laboratory for Special Fiber Photonic Devices, South China Normal University, Guangzhou, China

**Keywords:** binary coding, chiral metamaterial, photonic spin Hall effect, PT-symmetry

## Abstract

The photonic spin Hall effect (PSHE), featured by a spin-dependent shift driven by its polarization handedness, is proposed to facilitate the applications in precision metrology and quantum information processing. Here, due to the magnetoelectric coupling of the chirality, the PSHE is accompanied with Goos–Hänchen and Imbert–Fedorov effects. Taking advantage of this superiority, the transverse shift (TS) and longitudinal shift (LS) can be applied simultaneously. Rearranging the PT-symmetric scattering matrix, the responsive PSHE near the exceptional points and their basic physical mechanisms are discussed in detail in the case of complex chirality *κ*. Re[*κ*] and Im[*κ*] regulated the rich (at multi-angle), gaint (reach upper limit) and tunable (magnitude and direction) TS and LS, respectively. Based on the chirality-modulated PSHE, the novel applications in binary code conversion and barcode encryption are proposed systematically. By incorporating the quantum weak measurement technology, our applications provide new mechanisms to realize optoelectronic communication.

## 1 Introduction

The spin–orbit interaction of photons, corresponding to the interplay between the spin degree of freedom of light and the extrinsic orbital angular momentum, leads to a spin-dependent shift of light, namely the photonic version of the spin Hall effect [1, 2]. The photonic spin Hall effect (PSHE) will split the shifts of parallel and perpendicular to the incident plane of light simultaneously in some specific materials, such as anisotropy and two-dimensional materials [3–8]. The transverse shift (TS) splitting, implied by universal angular momentum conservation, is different from the Imbert–Fedorov (IF) shift, while the longitudinal shift (LS) occurs in the case of cross-polarization, which is also different from the Goos–Hänchen (GH) shift [9, 10]. Because the GH shift is polarization-dependent, which is described in terms of evanescent-wave penetration, while the LS is spin-dependent, it takes place as a result of an effective spin–orbit interaction [11, 12]. The interesting PSHE phenomena show promising applications in various realms, including imaging, precise metrogy and optical sensing [13–17]. However, the PSHE is generally weak and is always subwavelength magnitude, due to the weak spin–orbit interaction. Therefore, to enable and further extend these applications, manipulating a giant and controllable PSHE is desirable [18, 19]. In order to achieve that featured PSHE, many studies have been proposed by using the special properties of metamaterials, such as hyperbolic metamaterials [20, 21], parity-time (PT)-symmetric metamaterials [22, 23], epsilon-near-zero metamaterials, and chiral metamaterials, etc. [24–27].

Chiral metamaterials are composed of particles that cannot be superimposed with their mirror images using translation and rotation [28], which has different responses to a left circularly polarized (LCP) and a right circularly polarized (RCP) waves (eigenwaves of the wave equation in such media), due to the strong magnetoelectric coupling characteristic. The interesting and intriguing effects in chiral metamaterials are induced by circular dichroism (i.e., different absorption of LCP and RCP waves) and optical activity (i.e., the rotation of the polarization of an initially linearly polarized wave). We know that chirality appears in natural materials, but metamaterials will have stronger magnetoelectric coupling and huge effects. In 2004, Pendry discussed the possibility of negative refraction in chiral metamaterials [29]. Since then, chiral metamaterials have attracted much interest because of their features, such as negative refractive indices, asymmetric transmission, and broadband circular polarizers [30–32].

On the other hand, Bender and Boettcher’s pioneering work showed that a wide class of non-Hermitian Hamiltonians had an essentially real valued energy spectrum below the exceptional points (EPs) if they commute with the $\hat{P}\hat{T}$ operator, $\left[\hat{P}\hat{T},\hat{H}\right]=\hat{P}\hat{T}\hat{H}-\hat{H}\hat{P}\hat{T}=0$ [33]. Although the theoretical exploration of PT-symmetry originates from quantum mechanics, since the paraxial beam propagation is described by a Schrödinger-like equation, optics and photonics have proven to be the ideal platform for experimental observation and utilization of PT-symmetry, requiring the reactive index n*(**−r**) = *n*(**r**) [34]. The application of PT-symmetry in optics provides an opportunity to practically prove many unique and unusual phenomena, such as PT-breaking transitions, unidirectional invisibility and coherent perfect absorption (CPA)-laser [35–38].

When the beam interacts with matter, PSHE is a useful and intuitive metrological tool to characterize the physical properties of the structure. Although PSHE in PT-symmetric metamaterials and chiral metamaterials has attracted extensive research, respectively [22–25]. However, under the concept of PT-symmetry, an important class of metamaterials that has rarely been explored is the so-called chiral metamaterials [39, 40], let alone its PSHE. Compared with passive chirality, can circularly polarized waves preserve their handedness under the action of $\hat{P}$ and $\hat{T}$ operators in non-Hermitian system? How does their combination produce new properties and affect PSHE shifts? These problems and their included unique and unusual effects are still worth exploring. In consequence, such an undeveloped PT-symmetric chiral metamaterial should be potentially possible to yield a mass of entirely novel phenomena of PSHE and applications.

In this paper, the chirality-modulated PSHE in PT-symmetric system is studied and its new applications in photoelectric signal devices are explored. Firstly, using the newly arranged scattering matrix and the analytical solutions of PSHE shifts derived by angular spectrum method, we reveal the basic physical relationship between the variation law of the PT-phase and the PSHE distribution. Differently, both the magnitude and direction dimensions of the multiple PSHE can be modulated by Re[*κ*] and Im[*κ*], respectively. Interestingly, the similarities and differences between TS and LS are found: both LS and TS are symmetrically distributed with Re[*κ*] and are asymmetrically distributed with Im[*κ*]. Finally, its potential applications in binary code conversion and barcode encryption are presented completely.

## 2 Model and theory

Chiral media belong to a wide range of bi-isotropic media, which is characterized by the constitutive relations: **D** = *ɛɛ*_0_**E** + *i*(*κ*/*c*)**H** and **B** = *μμ*_0_**H** − *i*(*κ*/*c*)**E**, where *ɛ*, *μ* and *κ* refer to the relative permittivity, permeability and the chirality parameter (which quantifies the magnetoelectric coupling), respectively. *ɛ*_0_, *μ*_0_ are the vacuum permittivity and permeability, respectively, and *c* is the speed of light in vacuum. In PT-symmetrical system with chiral response, analogy to the Schrödinger equation in quantum mechanics, the eigenproblem of Maxwell’s equations ∇ × **E** = *iω***B** and ∇ × **H** = −*iω***D** is written in the form of $\bar{\bar{H}}T=\omega T$ , where $\bar{\bar{H}}$ is a pseudo-Hamiltonian tensor–operator and **T** is a generalized vector containing the fields. By solving **B** and **D** and following procedure of Refs. [40, 41], the necessary conditions for PT-symmetric chiral system can be obtained as:

(1) $$\varepsilon \left(r\right)=\varepsilon^{*}\left(-r\right),\;\mu \left(r\right)=\mu^{*}\left(-r\right),\;\kappa \left(r\right)=-\kappa^{*}\left(-r\right).$$

Then, we need to know the specific expressions of reflection and transmission coefficients of PT-symmetric chiral system before deriving the PSHE shifts. According to the continuity of the tangential components of **E** and **H** at the interfaces, by solving the 12 × 12 system of linear equations, we obtain the reflection and transmission coefficients of the transverse magnetic (TM) and transverse electric (TE) polarized incident waves, and the detailed processes refer to Ref. [39]. Due to the circular dichroism of the chiral metamaterials, the reflection and transmission coefficients of the circular polarizations can be obtained [42]:

(2) $$\begin{array}{cc} \left(\begin{array}{cc} t_{++} & t_{+-}\\ t_{-+} & t_{--} \end{array}\right) & =\frac{1}{2}\left(\begin{array}{cc} \left(t_{pp}+t_{ss}\right)-i\left(t_{ps}-t_{sp}\right) & \left(t_{pp}-t_{ss}\right)+i\left(t_{ps}+t_{sp}\right)\\ \left(t_{pp}-t_{ss}\right)-i\left(t_{ps}+t_{sp}\right) & \left(t_{pp}+t_{ss}\right)+i\left(t_{ps}-t_{sp}\right) \end{array}\right)\\ \left(\begin{array}{cc} r_{++} & r_{+-}\\ r_{-+} & r_{--} \end{array}\right) & =\frac{1}{2}\left(\begin{array}{cc} \left(r_{pp}-r_{ss}\right)-i\left(r_{ps}+r_{sp}\right) & \left(r_{pp}+r_{ss}\right)+i\left(r_{ps}-r_{sp}\right)\\ \left(r_{pp}+r_{ss}\right)-i\left(r_{ps}-r_{sp}\right) & \left(r_{pp}-r_{ss}\right)+i\left(r_{ps}+r_{sp}\right) \end{array}\right) \end{array}.$$

Because of two possible circularly polarized waves on both sides, the system can be described by four input and four output ports, so it can be given by a 4 × 4 description of scattering matrix, *S*, consisting of eight reflection and eight transmission amplitudes $\left\{r,t\right\}$ , as:

(3) $$\left[\begin{array}{c} N_{2}^{-}\\ M_{1}^{+}\\ N_{2}^{+}\\ M_{1}^{-} \end{array}\right]=S\left[\begin{array}{c} N_{1}^{-}\\ M_{2}^{+}\\ N_{1}^{+}\\ M_{2}^{-} \end{array}\right]\equiv \left[\begin{array}{cccc} t_{--}^{loss} & r_{-+}^{gain} & t_{-+}^{loss} & r_{--}^{gain}\\ r_{+-}^{loss} & t_{++}^{gain} & r_{++}^{loss} & t_{+-}^{gain}\\ t_{+-}^{loss} & r_{++}^{gain} & t_{++}^{loss} & r_{+-}^{gain}\\ r_{--}^{loss} & t_{-+}^{gain} & r_{-+}^{loss} & t_{--}^{gain} \end{array}\right]\left[\begin{array}{c} N_{1}^{-}\\ M_{2}^{+}\\ N_{1}^{+}\\ M_{2}^{-} \end{array}\right].$$

**Figure 1:**
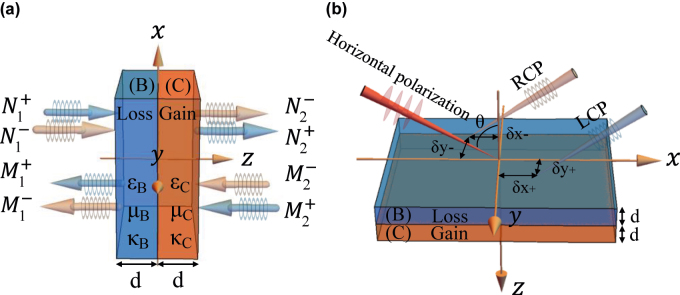
Schematic illustrating the wave transmission in the PT-symmetric chiral structure. (a) Diagram of the scattering of circularly polarized plane waves by a two-layer PT-symmetric chiral optical system. (b) Schematic of the LS and TS of the reflected PSHE. The loss layer *ɛ*_*B*_ = 9 + 1.5*i*, *μ*_*B*_ = 1 + 0.15*i*, *κ*_*B*_ = + Re[*κ*] + Im[*κ*]*i*. And the gain layer *ɛ*_*C*_ = 9 − 1.5*i*, *μ*_*C*_ = 1–0.15*i*, *κ*_*C*_ = − Re[*κ*] + Im[*κ*]*i*. d is the thickness of layers B and C.

In Eq. (3), $r_{++}^{loss}$ , $r_{--}^{loss}$ , $r_{+-}^{loss}$ , $r_{-+}^{loss}$ and $t_{++}^{loss}$ , $t_{--}^{loss}$ , $t_{+-}^{loss}$ , $t_{-+}^{loss}$ are the reflection and transmission coefficients for LCP(+)/RCP(−) light incident from the loss, while $r_{++}^{gain}$ , $r_{--}^{gain}$ , $r_{+-}^{gain}$ , $r_{-+}^{gain}$ and $t_{++}^{gain}$ , $t_{--}^{gain}$ , $t_{+-}^{gain}$ , $t_{-+}^{gain}$ are the reflection and transmission coefficients for LCP(+)/RCP(−) light incident from the gain side. The form of such a scattering matrix depends on the arrangement of the amplitudes $N_{1}^{\pm },N_{2}^{\pm },M_{1}^{\pm },M_{2}^{\pm }$ , where $N_{1}^{\pm },N_{2}^{\pm },M_{1}^{\pm }$ and $M_{2}^{\pm }$ are the amplitudes of the ingoing and outgoing RCP (−) and LCP (+) waves, as shown in Figure 1a. In order to appropriately illustrate the connection of the scattering matrix to the different input and output wave amplitudes, we adopt the above arrangement of the transmission and reflection coefficients. It satisfies the PT-symmetrical fundamental condition $\hat{P}\hat{T}S\left(\omega^{*}\right)\hat{P}\hat{T}=S^{-1}\left(\omega \right)$ and the generalized unitarity relation [35, 36]. Due to the importance of the PT-phases change on PSHE, we briefly introduce the PT-related phases and EPs. A surprising property is that the EPs for the TM and TE polarizations under oblique incidence are different. It means that there is an intermediate phase, called the mixed PT-phase, in which the PT-symmetric phase still exists for one polarization and is broken for the other polarization under the same conditions. For such a newly arranged matrix based on circular polarization (linear superposition of TM and TE waves), the mixed PT-phase is easily accessible. Indeed, calculating the eigenvalues of the scattering matrix in Eq. (3), we can get three different possible phases. Eigenvalues of the scattering matrix in full PT-symmetric phase, *σ*_*i*_, all are unimodular (i.e., obedience $\left|\sigma_{i}\right|=1$ ). While in fully PT broken-phase, $\left|\sigma_{i}\right|\neq 1$ , eigenvalues form pairs of reciprocal magnitude (subscript *i* = 1–4 represents different eigenvalues), and in mixed PT-phase, a pair of the eigenvalues is unimodular while the other is not ( $\left|\sigma_{1,2}\right|\neq 1$ and $\left|\sigma_{3,4}\right|=1$ ). At the symmetry breaking point, i.e., the EPs, two or more eigenvalues and eigenvectors coincide, making EPs to be singular points, associated with unique dynamic evolution characteristics.

Now a horizontally (H)-polarized Gaussian beam is illuminated on the PT-symmetric chiral structure, as shown in Figure 1b. Its angular spectrum is ${\tilde{E}}_{i}=\exp\left[-\left(k_{ix}^{2}+k_{iy}^{2}\right)w_{0}^{2}/4\right]{\hat{e}}_{ix}$ , where *w*_0_ = 100*λ* is the beam waist and *k*_*iy*_ (*k*_*ix*_) is the *y* (*x*) component of wave vector. *λ* is the wavelength of the incident wave. Under the paraxial approximation, the wavevector spread is small. Therefore, the Fresnel coefficients can be expanded into Taylor series with respect to *k*_*ix*_ [43–45]. The angular spectrum of the reflected beam relates to the incident beam through a transformation matrix, as is detailed in Ref. [7]. In the circular polarization basis, $e_{r\pm }=\left[e_{rx}\pm ie_{ry}\right]/\sqrt{2}$ , through the inverse Fourier transform of the reflected angular spectrum, the reflected field in real space at *z*_*r*_ > 0 can be obtained:

(4) $$\begin{array}{ll} E_{r\pm }\left(x_{r},y_{r}\right) & =\left\{\left[r_{pp}-x_{r}\zeta r_{pp}^{\prime }+y_{r}\zeta U\right]\mp i\left[r_{sp}-x_{r}\zeta r_{sp}^{\prime }\right.\right.\\ & \;\left.\left.-y_{r}\zeta V\right]\exp\left[\frac{k_{0}\zeta \left(x_{r}^{2}+y_{r}^{2}\right)}{-2i}\right]\right\}e_{r\pm }. \end{array}$$

Here, *ζ* = *i*/(*z*_*R*_ + *iz*_*r*_), *U* = (*r*_*sp*_ − *r*_*ps*_) cot *θ*, *V* = (*r*_*pp*_ + *r*_*ss*_) cot *θ*, Rayleigh length $z_{R}=k_{0}w_{0}^{2}/2$ , and $r_{pp}^{\prime }$ $\left(r_{sp}^{\prime }\right)$ is the first derivative of *r*_pp_ (*r*_sp_) with respect to the incident angle *θ*. *x*_*r*_, *y*_*r*_ and *z*_*r*_ are the coordinate components of Cartesian coordinate system at the reflected path. Spin–orbit interaction occurs during reflection: spin angular momentum in normal direction varies with beam reflection, so that it must be compensated by the PSHE shift resulting in a nonzero extrinsic orbital angular momentum, owing to the conservation of total angular momentum in the normal direction. With respect to the geometric prediction, the centroid shifts of LCP and RCP can be defined as [2]:

(5) $$\left[\delta_{x\pm },\delta_{y\pm }\right]=\frac{\iint \left[x_{r},y_{r}\right]{\left|E_{r\pm }\left(x_{r},y_{r}\right)\right|}^{2}dx_{r}dy_{r}}{\iint {\left|E_{r\pm }\left(x_{r},y_{r}\right)\right|}^{2}dx_{r}dy_{r}}.$$

After some mathematical calculations, we obtain:

(6) $$\begin{array}{ll} \delta_{x\pm } & =\left\{Im\left[r_{pp}^{*}r_{pp}^{\prime }+r_{sp}^{*}r_{sp}^{\prime }\right]\right.\\ & \;\left.\pm Re\left[+r_{sp}^{*}r_{pp}^{\prime }-r_{pp}^{*}r_{sp}^{\prime }\right]\right\}/k_{0}W_{\pm } \end{array}$$

(7) $$\begin{array}{ll} \delta_{y\pm } & =\left\{-Im\left[r_{pp}^{*}U-r_{sp}^{*}V\right]\right.\\ & \;\left.\pm Re\left[-r_{sp}^{*}U-r_{pp}^{*}V\right]\right\}/k_{0}W_{\pm }, \end{array}$$

where

(8) W±=rpp2+rsp2±2Imrpp*rsp+1k02w02rpp′2+rsp′2±2Imrpp′*rps′ +|U|2+|V|2∓2ImU*V.

Both *δ*_*x*±_ and *δ*_*y*±_ contain two terms. The first term of *δ*_*x*±_ is the conventional GH shift, while the first term of *δ*_*y*±_ is the IF shift. They are spin-independent shifts, moving the RCP and LCP components of the reflected beam together. The second terms of *δ*_*x*±_ and *δ*_*y*±_ are spin-dependent, originating from the spin–orbit interaction. The cooperation effect of the spin-independent and spin-dependent terms of the centroid shifts will result in more abundant splitting. RCP and LCP components will shift toward opposite directions in these terms. Here, the non-diagonal reflection coefficients *r*_sp_ and *r*_ps_ play an important role in the spin–orbit interaction of light [24]. In other words, due to the magnetoelectric coupling caused by chirality, the conversion of intrinsic spin angular momentum and extrinsic orbital angular momentum on the surface of PT-symmetric chiral metamaterial can be modulated by the chirality. These characteristics will provide theoretical support for the following discussion of rich PSHE phenomena and the related applications.

## 3 Results and discussion

**Figure 2:**
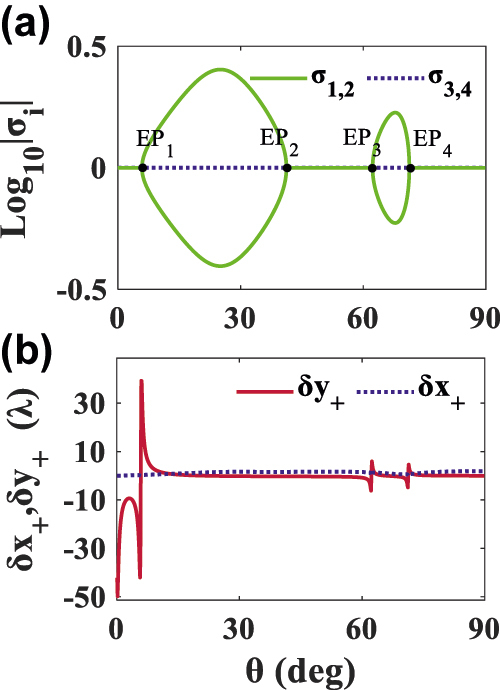
Wave scattering and its PSHE in PT-symmetric bilayer. (a) Logarithm of the modulus of the eigenvalues of the scattering matrix (*σ*_1,2_ and *σ*_3,4_) as a function of incident angle *θ* based on circular polarization in the specific case of *κ* = 0. EP_1_, EP_2_, EP_3_, and EP_4_ are the exceptional points. (b) The corresponding longitudinal shift *δ*_*x*+_ (blue dotted line) and transverse shift *δ*_*y*+_ (red solid line) under the incidences.

In PT-symmetry system, a large amount of work concerns scattering configurations rather than paraxial beam propagation systems to discuss the PT-phase change and the singularity of the EPs. We make the dimensionless frequency *ωd*/*c* = 6.5 and assume the simplest case chirality *κ* = 0 first. Note that in this case the *S* matrice is $\left[\begin{array}{cc} t & r^{\text{gain}}\\ r^{\text{loss}} & t \end{array}\right]$ , as defined in Ref. [46]. Since the TM and TE waves have different EPs, the eigenstates, corresponding to the eigenvalues of the four-channel scattering matrix, can be explained more conveniently based on circular polarization. Thus, the relationship between the eigenvalues of the scattering matrix and PSHE is discussed to clarify the most basic physical mechanism of the PSHE, as shown in Figure 2. Evidently, because of the vanishment of the nondiagonal reflection coefficients *r*_ps_ and *r*_sp_, the system will degenerate into a pure PT-symmetric system that only balances *ɛ* and *μ*. Here, interestingly, different from the previous discussion, the broken phase disappears and the mixed phase arises [23]. The *δ*_*y*_ is zero at EPs, but its absolute is largely enhanced in the vicinities of EP_1_, EP_3_, and EP_4_. The maximum value of TS could reach −42*λ* at 5.7° near EP_1_.

**Figure 3:**
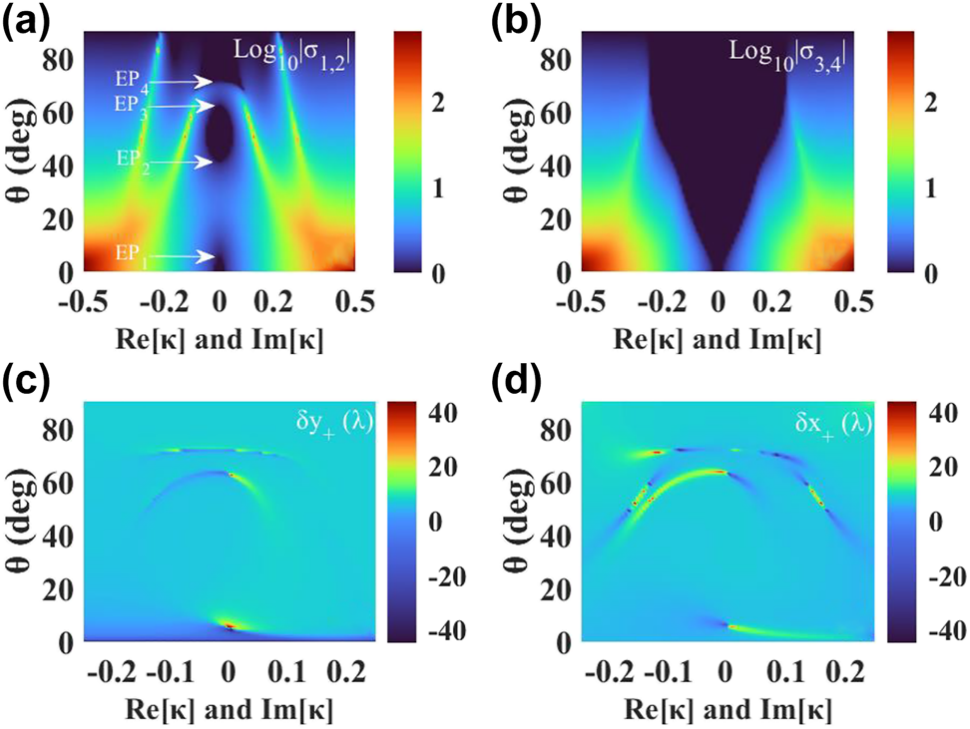
Wave scattering and the PSHE under arbitrary complex chirality. (a) and (b) Eigenvalues of the scattering matrix in Eq. (3) change with both real (Re[*κ*]) and imaginary (Im[*κ*]) parts of chirality *κ* and incident angle *θ*. (c) and (d) The corresponding distribution of *δ*_*y*+_ and *δ*_*x*+_ with both Re[*κ*] and Im[*κ*] and incident angle *θ*, separately. Here *κ*_*B*_ = +Re[*κ*] + Im[*κ*]*i*, *κ*_*C*_ = −Re[*κ*] + Im[*κ*]*i*.

The solutions of the scattering matrix *S*, i.e., *σ*_1,2_ and *σ*_3,4_, in Figure 3a and Figure 3b, are distributed with incident angle *θ* and both Re[*κ*] and Im[*κ*] (*κ* = ±Re[*κ*] + Im[*κ*]*i*). In the full PT-symmetric phase, the energy eigenvalues are real and the circular polarizations are preserved without attenuation and amplification ( ${Log}_{10}\left|\sigma_{1,2}\right|=0$ and ${Log}_{10}\left|\sigma_{3,4}\right|=0$ ). In the mixed PT-phase, a pair of circular polarizations remain, while the other change ( ${Log}_{10}\left|\sigma_{1,2}\right|\neq 0$ and ${Log}_{10}\left|\sigma_{3,4}\right|=0$ ), and in the fully broken PT-phase, the energy become complex, and the circular polarizations are attenuated or amplified ( ${Log}_{10}\left|\sigma_{1,2}\right|\neq 0$ and ${Log}_{10}\left|\sigma_{3,4}\right|\neq 0$ ). The critical curves of full PT-phase and mixed PT-phase are the EPs, which has been marked on the Figure 3a. In the range of Re[*κ*] and Im[*κ*] from −0.1 to 0.1, by comparing the eigenvalues of the scattering matrix of Figures 3a and b, when the Re[*κ*] and Im[*κ*] are fixed and *θ* increases, the system passes from PT-symmetric phase to mixed PT-phase, then re-enters to PT-symmetric phase and with further increase of *θ* re-enters to mixed PT-phase and, finally, ends up in the fully PT-symmetric phase.

Figures 3c and d show the distribution of *δ*_*y*_ and *δ*_*x*_ of LCP light with incident angle *θ* and both Re[*κ*] and Im[*κ*]. *δ*_*y*_ is not completely symmetrical distributed in the range of 6°, 45–62°, and 72° of Re[*κ*] and Im[*κ*] from −0.1 to 0.1. Combined with the variation law of PT-phase distribution, it can be known that the large *δ*_*y*_ are distributed near EP_1_, EP_3_, and EP_4_ curves. It is worth noting that the sign of shifts near EP_3_ is switchable with the chirality *κ*, which is another PSHE regulation method in dimension of direction besides its magnitude. Here, the reason for the large *δ*_*y*_ is that $\left|r_{pp}\right|=\left|r_{sp}\right|=\left|r_{ps}\right|\neq 0$ near the EPs, which is the most intuitive manifestation of rich PSHE caused by the occurrence of the chirality. Interestingly, it can be seen from the combination of Figures 3c and d that the distributions of *δ*_*y*_ and *δ*_*x*_ are roughly similar. However, due to the $r_{sp}^{\prime }$ and $r_{pp}^{\prime }$ on the numerator of Eq. (6), the different behaviors of *δ*_*x*_ occur.

Whether the *δ*_*y*_ or *δ*_*x*_, the underlying physics is associated with the near-zero absolute value and abrupt phase jump of the *r*_pp_ near the EPs. Through the analysis, the giant and switchable PSHE can be obtained by independently adjusting the chirality *κ* to change the position of EPs, other than *ɛ* and *μ*. This finding represents direct relevance of PSHE related to spontaneous PT-symmetry breaking in PT-symmetric chiral system.

**Figure 4:**
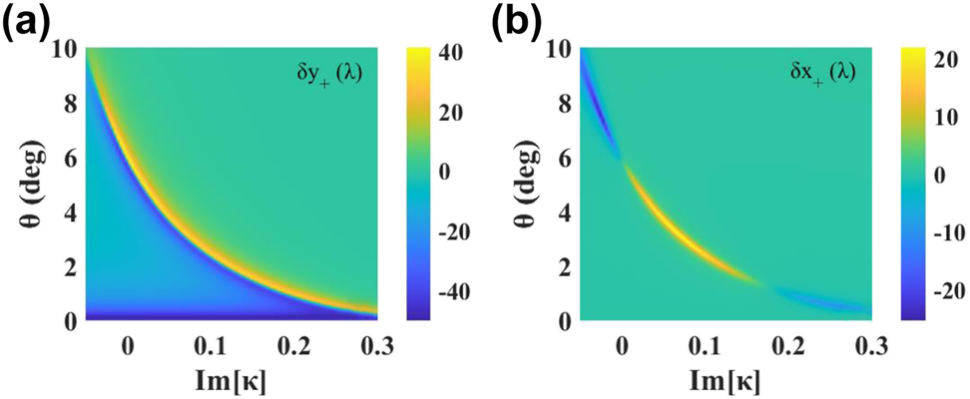
Distribution of (a) *δ*_*y*+_ and (b) *δ*_*x*+_ with chirality Im[*κ*] and incident angle *θ* in the case of Re[*κ*] = 0, respectively.

For detailed discussion, we further discuss the effect of only Im[*κ*] (Re[*κ*] = 0) on PSHE. As shown in Figure 4, with the change of weak chirality Im[*κ*] from −0.05 to 0.3, the distribution of TS and LS is still similar. In general, we know that there are two methods to enhance the spin splitting of light, according to: $\delta_{y\pm }=\mp \frac{cot\theta }{k_{0}}Re\left[1+\frac{r_{s}}{r_{p}}\right]$ [47–49]. A general strategy of a large number of research reports for enhancing such a shift is to achieve a large ratio $\left|r_{s}\right|/\left|r_{p}\right|$ at Brewster angle [50]. Slightly different from the previous report, the large TS still occurs at $\left|r_{pp}\right|=\left|r_{sp}\right|=\left|r_{ps}\right|\neq 0$ here. For example, the TS reaches −41.5*λ* at 6.5° when Im[*κ*]=−0.01 (see Figure S1). Another enhancement strategy of PSHE can be achieved by increasing cot*θ*. We know that cot*θ* approaches infinite as *θ* reduces to zero. This kind of PSHE enhancement at a very small incident angle can be well reflected in the dark blue area at the bottom of Figure 4a. Here, TS reaches the upper limit 50*λ* near 0.2°, because the beam waist determines the upper limit of the spin shift. In fact, the TS always has $\left|\delta_{y}\right|\leq w_{0}/2$ [51]. The reports of enhancement in this near-normal incident mode have been proved experimentally recently [52, 53]. Their underlying mechanisms may be well explained by the vector varying Pancharatnam–Berry phase, which triggers a phase transition from vortex generation to spin-Hall shift [54, 55].

Similarly, the situation of *δ*_*x*_ is like the above analysis, excepting that there is no large shift at the bottom of Figure 4b, because there are no *U* and *V* terms on the numerator of Eq. (6). According to the necessary conditions of PT-symmetric chiral system, layers B and C have the same chirality value in this case, which makes the PSHE varies asymmetrically with Im[*κ*] for both *δ*_*y*_ and *δ*_*x*_. In general, by adjusting the Im[*κ*] in a PT-symmetric chiral system, two strategies can be achieved simultaneously to obtain a large shift.

**Figure 5:**
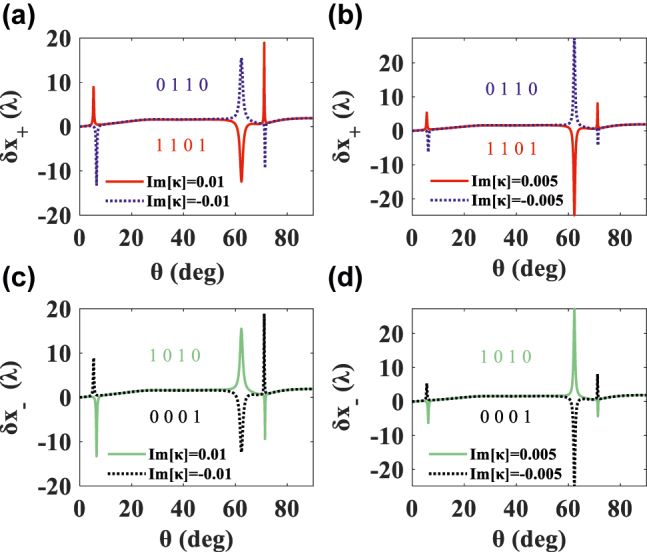
LS of LCP and RCP light versus incident angle *θ* with Re[*κ*] = 0. Encoding “four-digit barcode A”: “1 1 0 1” (positive Im[*κ*]) and “0 1 1 0” (negative Im[*κ*]) for the LCP light. Realizing “1 0 1 0” (positive Im[*κ*]) and “0 0 0 1” (negative Im[*κ*]) for the RCP component.

When exploring the LS distribution in Figure 4b, in addition to the small angle, there are two large shifts near 62.3° and 71.2°, due to spontaneous PT-symmetry breaking, as shown in Figure 5. Obviously, the sign of LS corresponds inversely to the sign of the Im[*κ*] near 62.3°, while near 71.2°, it corresponds to the sign of the Im[*κ*]. Therefore, we can control the Im[*κ*] to obtain a positive or negative LS near 62.3° and 71.2°. This switchable PSHE phenomenon has practical applications. For example, in terms of materials, we control the positive or negative chirality of materials and use it as polarizing devices and optical switches, etc. Conversely, in optical field, the PSHE can be observed to analyze the properties and physical parameters of materials, including studying the exotic physics near the EPs in various photonic systems.

Here, differently, we propose two novel functions of implementing two-digit binary code conversion and four-digit barcode encryption by controlling the chirality parameter and observing its responsive PSHE (only LS is discussed here, and the TS discussion is placed in the Supplementary Material). First, we set our coding principle: the positive (negative) of Im[*κ*] or Re[*κ*] represents “1” (“0”), and the LCP (RCP) light represents “1” (“0”). These two constitute the binary parametric input code [chirality, polarization]. The positive (negative) of the second LS represents “1” (“0”), similarly, and the positive (negative) of the third LS represents “1” (“0”). These two phenomena form the observational output code [sign of the second LS, sign of the third LS]. Since the system is a four independent channel system, two binary code groups will combine into a “four-digit barcode A”, based on the *δ*_*x*_: “chirality, polarization, sign of the second LS, sign of the third LS”. And “four-digit barcode B” based on *δ*_*y*_ is placed in Figure S1, using the same method.

We then illustrate the two-digit code conversion process: when analyze the LCP light and Im[*κ*] > 0, the input code is [1, 1], as shown in red line of Figure 5. It can be observed that the LS is negative at 62.3° and positive at 71.2°, so the output code is [0, 1]. By analyzing the LCP light with negative Im[*κ*] in blue line of Figure 5, the input code is [0, 1], then the output code is [1,0], according to the phenomenon of positive LS at 62.3° and negative value at 71.2°. Similarly, in the second line of Figure 5, the input code of green (black) line is [1, 0] ([0, 0]) and the output code is [1, 0] ([0, 1]). The encryption system of “four-digit barcode A” has been marked in Figure 5. “four-digit barcode B” based on the TS can be obtained “1 0 1 1” and “1 1 1 1” additionally. In this way, we obtained two sets of A and B barcodes.

**Figure 6:**
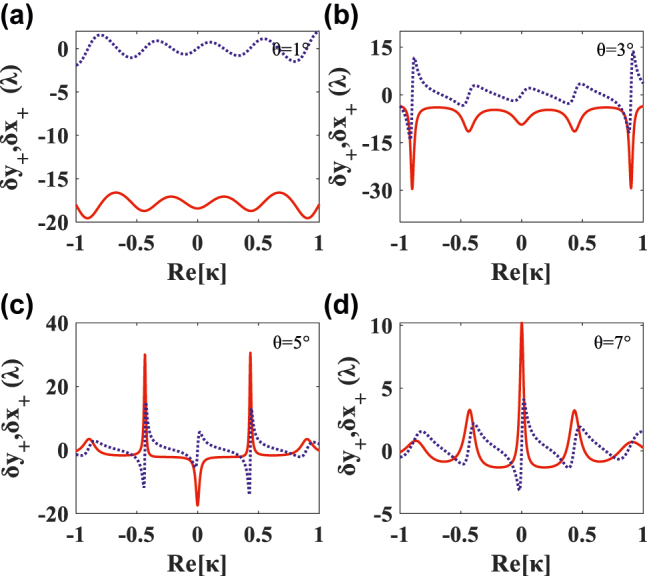
In the absence of Im[*κ*], the dependences of the *δ*_*y*+_ (red solid line) and *δ*_*x*+_ (blue dotted line) on the Re[*κ*] at four different small incident angles: (a) *θ* = 1°, (b) *θ* = 3°, (c) *θ* = 5°, and (d) *θ* = 7°.

The TS and LS are asymmetrically distributed with the Im[*κ*] as discussed ahead. On the contrary, in terms of Re[*κ*] change (Im[*κ*] = 0), the TS is axisymmetrically and the LS is centrosymmetrically distributed with Re[*κ*], as shown in Figure 6. With the increase of incident angle *θ*, the number of shift peaks does not change for both *δ*_*y*_ and *δ*_*x*_. The reason for the symmetry of the PSHE spectrum is that the Re[*κ*] of layers B and C have the same value but opposite signs. Unlike the previous discussion, the maximum of TS and LS occurs at $\left|r_{pp}\right|=\left|r_{sp}\right|=\left|r_{ps}\right|=0$ here. It can be seen from the Figure 6 that not only the negative TS can be obtained by controlling the incident angle, for example, all TS are negative at 1° and 3°, but also a large positive TS can be obtained, up to 30.6*λ* at 5°, by controlling chirality Re[*κ*] = 0.43. In short, besides adjusting the incident angle, we can also fix *θ* and obtain huge positive or negative PSHE by regulating the Re[*κ*].

**Figure 7:**
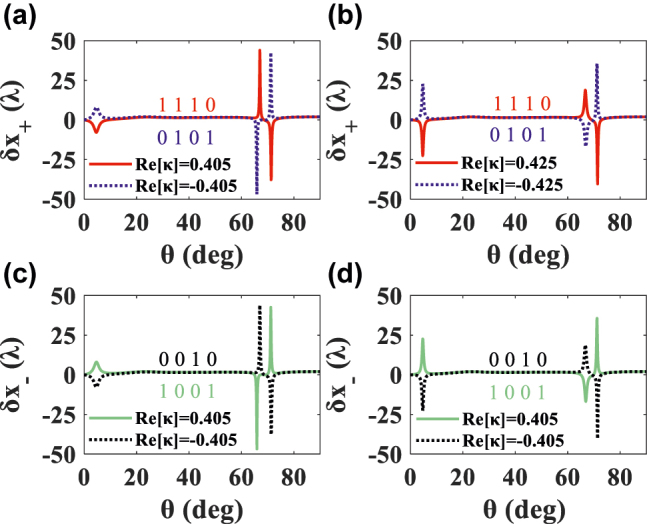
LS of LCP and RCP components changing with the incident angle *θ* in the case of Im[*κ*] = 0. The implementation of “four-digit barcode A”: “1 1 1 0” (positive Re[*κ*]) and “0 1 0 1” (negative Re[*κ*]) for the LCP. Encoding “1 0 0 1” (positive Re[*κ*]) and “0 0 1 0” (negative Re[*κ*]) for the RCP light.

As can be seen from Figure 7, when the Re[*κ*] is positive (negative), the LS is positive (negative) at 67° and negative (positive) at 71.3°, which is opposite to the change of LS with Im[*κ*]. The process of Re[*κ*] modulated PSHE to realize code conversion and barcode encryption is discussed below. Obviously, when the Re[*κ*] is positive (negative), the input code of the LCP light is [1, 1] ([0, 1]), showing the output code [1, 0] ([0, 1]) that LS is positive (negative) at 67° and negative (positive) at 71.3°, such as the red (blue) line in Figure 7. In addition, it is easy to find that the input and output codes of green (black) line are [1, 0] ([0, 0]) and [0, 1] ([1, 0] [0, 1]), for RCP. Their combination “four-digit barcode A” has been marked in Figure 7. In addition, “1 1 0 0” and “1 0 0 0” can be obtained based on *δ*_*y*_, in Figure S2.

Binary coding is easy to implement in technology and simple in operation rules, which is widely used in the field of computer technology and communication. In terms of binary code conversion function, only two output codes [1, 0] [0, 1] are modulated by adjusting the Re[*κ*] and Im[*κ*] based on *δ*_*x*_. However, all four input and output codes [0, 0], [0, 1], [1, 0] [0,1], and [1, 1] are perfectly completed based on *δ*_*y*_. In the barcode encryption function, two sets of A and B barcodes form a very comprehensive barcode encryption system. Based on the above analysis, in the PT-symmetric chiral system, by adjusting the Re[*κ*] and Im[*κ*] and observing *δ*_*x*_ and *δ*_*y*_, two functions of binary four-digit barcode encryption and two-digit code conversion are realized simultaneously, which provides positive significance for photoelectric communication encryption and photoelectric integration system.

**Figure 8:**
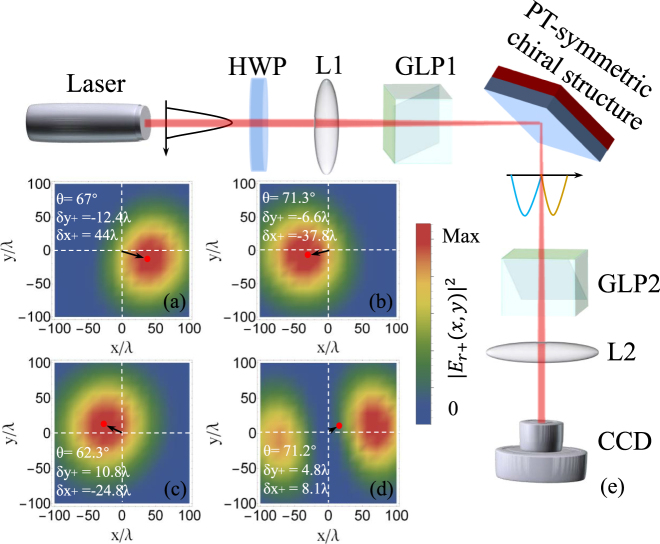
The possible experimental scheme and the realization of barcode encryption. (a–d) The intensity distribution of the LCP reflected light and (e) experiment setup of the quantum weak measurement system scheme. (a) and (b) Re[*κ*] = 0.405, Im[*κ*] = 0; (c) and (d) Re[*κ*] = 0, Im[*κ*] = 0.005. HWP: Half-wave plate; GLP1 and GLP2: Glan laser polarizer; QWP: Quarter-wave plate; L1 and L2: Lens; CCD: Charge-coupled-device.

Finally, it is concluded that chirality modulates the rich, giant LS and TS. The observation of PSHE shifts by quantum weak measurement technology has been maturely realized in experiments. In order to demonstrate the feasibility of code conversion and barcode encryption in the experiment, we propose the possible experimental scheme of PSHE in PT-symmetric chiral system, as shown in Figure 8. The Gaussian beam generated by the laser enters the quantum weak measurement system in Figure 8e. The centroid shifts of the LCP reflected beam can be observed through the intensity distribution recorded by CCD, and the corresponding encrypted barcode can be read, as shown in Figure 8a–d. Combine Figures 8a and b, “four-digit barcode A”: “1 1 1 0”, “four-digit barcode B”: “1 1 0 0”. Figures 8c and d, “four-digit barcode A”: “1 1 0 1” and “four-digit barcode B”: “1 1 1 1”.

## 4 Conclusions

In this paper, the novel chirality-modulated PSHE and its applications in code conversion and barcode encryption have been studied in the PT-symmetric chiral system. After deriving the analytical PSHE shifts and discussing the PT-phase change distribution law, we revealed that the huge PSHE shifts occur near the EPs. In detail, the large and tunable TS and LS can be obtained not only by incident angle, but also by Re[*κ*] and Im[*κ*]. The similarities and the differences between TS and LS are revealed. LS and TS are symmetrically distributed with Re[*κ*] and are asymmetrically distributed with Im[*κ*]. Based on these PSHE phenomena, the intact code conversion and barcode encryption functions of the system are obtained. Finally, by incorporating the quantum weak measurement technology, the applications in photoelectric signal processing are put forward, which opens up the prospect for novel and adjustable photoelectric devices.

## Supplementary Material

Supplementary Material Details

## References

[1] Bliokh K., Aiello A. (2013). Goos–Hänchen and Imbert–Fedorov beam shifts: An overview. J. Opt..

[2] Ling X., Zhou X., Huang K. (2017). Recent advances in the spin Hall effect of light. Rep. Prog. Phys..

[3] Zhang W., Wu W., Chen S. (2018). Photonic spin Hall effect on the surface of anisotropic two-dimensional atomic crystals. Photon. Res..

[4] Zhang W., Wang Y., Chen S., Wen S., Luo H. (2022). Photonic spin Hall effect in twisted few-layer anisotropic two-dimensional atomic crystals. Phys. Rev. A.

[5] Liu M., Cai L., Chen S., Liu Y., Luo H., Wen S. (2017). Strong spin-orbit interaction of light on the surface of atomically thin crystals. Phys. Rev. A.

[6] Cai L., Liu M., Chen S. (2017). Quantized photonic spin Hall effect in graphene. Phys. Rev. A.

[7] Tang T., Li J., Luo L., Sun P., Yao J. (2018). Magneto-optical modulation of photonic spin Hall effect of graphene in terahertz region. Adv. Opt. Mater..

[8] Lin H., Chen B., Yang S. (2018). Photonic spin Hall effect of monolayer black phosphorus in the Terahertz region. Nanophotonics.

[9] Zhou X., Liu S., Ding Y., Min L., Luo Z. (2019). Precise control of positive and negative Goos–Hänchen shifts in graphene. Carbon.

[10] Zhen W., Deng D. (2020). Goos–Hänchen shifts for Airy beams impinging on graphene-substrate surfaces. Opt. Express.

[11] Qin Y., Li Y., Feng X., Xiao Y., Yang H., Gong Q. (2011). Observation of the in-plane spin separation of light. Opt. Express.

[12] Bliokh K., Rodríguez-Fortuño F., Nori F., Zayats A. (2015). Spin–orbit interactions of light. Nat. Photonics.

[13] Zhu T., Lou Y., Zhou Y. (2019). Generalized spatial differentiation from the spin Hall effect of light and its application in image processing of edge detection. Phys. Rev. Applied.

[14] Zhou X., Xiao Z., Luo H., Wen S. (2012). Experimental observation of the spin Hall effect of light on a nanometal film via weak measurements. Phys. Rev. A.

[15] Zhou X., Ling X., Luo H., Wen S. (2012). Identifying graphene layers via spin Hall effect of light. Appl. Phys. Lett..

[16] Zhu W., Xu H., Pan J. (2020). Black phosphorus terahertz sensing based on photonic spin Hall effect. Opt. Express.

[17] Liang C., Wang G., Deng D., Zhang T. (2021). Controllable refractive index sensing and multi-functional detecting based on the spin Hall effect of light. Opt. Express.

[18] Kim M., Lee D., Ko B., Rho J. (2020). Diffraction-induced enhancement of optical spi Hall effect in a dielectric grating. APL Photon..

[19] Kim M., Lee D., Kim Y., Rho J. (2022). Generalized analytic formula for spin Hall effect of light: Shift enhancement and interface independence. Nanophotonics.

[20] Kapitanova P., Ginzburg P., Rodríguez-Fortuño F. (2014). Photonic spin Hall effect in hyperbolic metamaterials for polarization-controlled routing of subwavelength modes. Nat. Commun..

[21] Kim M., Lee D., Kim T., Yang Y., Park H., Rho J. (2019). Observation of enhanced optical spin Hall effect in a vertical hyperbolic metamaterial. ACS Photonics.

[22] Fu Y., Fei Y., Dong D., Liu Y. (2019). Photonic spin Hall effect in PT symmetric metamaterials. Front. Physiol..

[23] Zhou X., Lin X., Xiao Z. (2019). Controlling photonic spin Hall effect via exceptional points. Phys. Rev. B.

[24] Wang H., Zhang X. (2011). Unusual spin Hall effect of a light beam in chiral metamaterials. Phys. Rev. A.

[25] Xu G., Zang T., Mao H., Pan T. (2011). Transverse shifts of a reflected light beam from the air-chiral interface. Phys. Rev. A.

[26] Chen H., Guan D., Zhu W. (2021). High-performance photonic spin Hall effect in anisotropic epsilon-near-zero metamaterials. Opt. Lett..

[27] Luo H., Wen S., Shu W., Tang Z., Zou Y., Fan D. (2009). Spin Hall effect of a light beam in left-handed materials. Phys. Rev. A.

[28] Lindell I., Sihvola A., Tretyakov S., Viitanen A. (1994). Electromagnetic Waves in Chiral and Bi-isotropic Media.

[29] Pendry J. B. (2004). A chiral route to negative refraction. Science.

[30] Plum E., Zhou J., Dong J. (2009). Metamaterial with negative index due to chirality. Phys. Rev. B.

[31] Menzel C., Helgert C., Rockstuhl C. (2010). Asymmetric transmission of linearly polarized light at optical metamaterials. Phys. Rev. Lett..

[32] Gansel J., Thiel M., Rill M. (2009). Gold helix photonic metamaterial as broadband circular polarizer. Science.

[33] Bender C., Boettcher S. (1998). Real spectra in non-hermitian Hamiltonians having PT symmetry. Phys. Rev. Lett..

[34] Miri M., Alù A. (2019). Exceptional points in optics and photonics. Science.

[35] Chong Y., Ge L., Stone A. (2011). PT-symmetry breaking and laser-absorber modes in optical scattering systems. Phys. Rev. Lett..

[36] Ge L., Chong Y., Stone A. (2012). Conservation relations and anisotropic transmission resonances in one-dimensional PT-symmetric photonic heterostructures. Phys. Rev. A.

[37] Lin Z., Ramezani H., Eichelkraut T., Kottos T., Cao H., Christodoulides D. (2011). Unidirectional invisibility induced by PT-symmetric periodic structures. Phys. Rev. Lett..

[38] Sun Y., Tan W., Li H., Li J., Chen H. (2014). Experimental demonstration of a coherent perfect absorber with PT phase transition. Phys. Rev. Lett..

[39] Katsantonis I., Droulias S., Soukoulis C., Economou E., Kafesaki M. (2020). PT-symmetric chiral metamaterials: asymmetric effects and PT-phase control. Phys. Rev. B.

[40] Droulias S., Katsantonis I., Kafesaki M., Soukoulis C., Economou E. (2019). Chiral metamaterials with PT symmetry and beyond. Phys. Rev. Lett..

[41] Castaldi G., Savoia S., Galdi V., Alù A., Engheta N. (2013). PT metamaterials via complex-coordinate transformation optics. Phys. Rev. Lett..

[42] Lekner J. (1996). Optical properties of isotropic chiral media. Pure Appl. Opt..

[43] Pan M., Li Y., Ren J. (2013). Impact of in-plane spread of wave vectors on spin Hall effect of light around Brewster’s angle. Appl. Phys. Lett..

[44] Ren J., Wang B., Xiao Y., Gong Q., Li Y. (2015). Direct observation of a resolvable spin separation in the spin Hall effect of light at an air-glass interface. Appl. Phys. Lett..

[45] Ren J., Wang B., Pan M., Xiao Y., Gong Q., Li Y. (2015). Spin separations in the spin Hall effect of light. Phys. Rev. A.

[46] Feng L., Xu Y., Fegadolli W. (2013). Experimental demonstration of a unidirectional reflectionless parity-time metamaterial at optical frequencies. Nat. Mater..

[47] Kim M., Lee D., Rho J. (2021). Spin Hall effect under arbitrarily polarized or unpolarized light. Laser Photon. Rev..

[48] Kim M., Lee D., Nguyen T., Lee H., Byun G., Rho J. (2021). Total reflection-induced efficiency enhancement of the spin Hall effect of light. ACS Photonics.

[49] Kim M., Lee D., Cho H., Min B., Rho J. (2021). Spin Hall effect of light with near-unity efficiency in the microwave. Laser Photon. Rev..

[50] Ling X., Xiao W., Chen S., Zhou X., Luo H., Zhou L. (2021). Revisiting the anomalous spin-Hall effect of light near the Brewster angle. Phys. Rev. A.

[51] Kim M., Lee D., Rho J. (2022). Incident-polarization-independent spin Hall effect of light reaching half beam waist. *Laser Photon. Rev.*.

[52] Ling X., Guan F., Cai X. (2021). Topology-induced phase transitions in spin-orbit photonics. Laser Photon. Rev..

[53] Kim M., Lee D., Yang Y., Kim Y., Rho J. (2022). Reaching the highest efficiency of spin Hall effect of light in the near-infrared using all-dielectric metasurfaces. Nat. Commun..

[54] Ling X., Guan F., Zhang Z., Xu H., Xiao S., Luo H. (2021). Vortex mode decomposition of the topology-induced phase transitions in spin-orbit optics. Phys. Rev. A.

[55] Ling X., Luo H., Guan F., Zhou X., Luo H., Zhou L. (2020). Vortex generation in the spin-orbit interaction of a light beam propagating inside a uniaxial medium: origin and efficiency. Opt. Express.

