# Supplementary material for: Chirality-modulated photonic spin Hall effect in PT-symmetry
Source: Nanophotonics. 2022 Jun 28;11(15):3475–84. doi: 10.1515/nanoph-2022-0229 (PMC11501175; doi:10.1515/nanoph-2022-0229)
Supplement: Supplementary file 1 — Supplementary Material Details [file j_nanoph-2022-0229_suppl.pdf]

Research Article

Chengkang Liang, Dongxue Liu, Rao Liu, Dongmei Deng, and Guanghui Wang*

Supplementary Material: Chirality-modulated photonic spin Hall effect in PT-symmetry

https://doi.org/10.1515/sample-YYYY-XXXX
Received Month DD, YYYY; revised Month DD, YYYY; accepted Month DD, YYYY

1 Code conversion and barcode encryption B based on transverse shift

Due to the difference between longitudinal shift (LS) and transverse shift (TS), we discuss the binary coding conversion and "four-digit barcode B" functions based on the TS. First, we clarify the coding principle based on TS: the positive (negative) of Im[κ] or Re[κ] represents "1" ("0"), and the LCP (RCP) light represents "1" ("0"). These two constitute the binary parametric input code [chirality, polarization]. The positive (negative) of the second TS represents "1" ("0"), while the third TS that (do not) crosses the 0-threshold represents ("0") "1". Similarly, these two phenomena form the binary observational output code [sign of the second TS, 0-threshold of the third TS]. And the "four-digit barcode B" based on δ_y are: "chirality, polarization, sign of the second TS, 0-threshold of the third TS".

When we analyze the LCP light with Im[κ]>0, the input code is [1,1], as shown in the red line of **Figure S1**. It can be observed that the TS is positive at 62.3° and crosses the 0-threshold values at 71.2°, so the output code is [1,1]. By analyzing the blue line of the LCP light with negative Im[κ] in the first line of Figure S1, the input code is [0,1], then the output code is [0,1], according to the phenomenon of negative TS at 62.3° and crossing 0-threshold value at 71.2°. Similarly, in the second line, the input code of green (black) line is: [1,0] ([0,0]) and the output code is [1,1] ([0,1]). Their combination "four-digit barcode B" are marked in Figure S1.

The sign of Im[κ] change is corresponding to the sign of TS near 62.3°, however, the difference is that the sign of Re[κ] change is opposite to the sign of TS. In detail, when the Re[κ] is positive (negative), the TS is negative (positive) at both 67° and 71.3°. By analogy with the effect of Im[κ] on PSHE, the Re[κ] regulation to realize code conversion and barcode encryption is discussed below. Obviously, when the Re[κ] is positive (negative), the input code of the LCP light is [1,1] ([0,1]), showing that the TS is negative (positive) and does not cross the 0-threshold output code [0,0] ([1,0]), such as the red (blue) line in **Figure S2**. In addition, it is easy to find that the green and black lines input codes and output codes are [1,0], [0,0] and [0,0], [1,0] respectively, for RCP light. And their combination "four-digit barcode B" are also marked in Figure S2.

In terms of binary code conversion function, two binary codes of [0,0] [1,0] are modulated by adjusting the Re[κ]. In addition to the [0,1] [1,1] realized by the above adjustment of Im[κ], all four input codes and

***Corresponding author: Guanghui Wang,** Guangdong Provincial Key Laboratory of Nanophotonic Functional Materials and Devices, South China Normal University, Guangzhou, China; Guangzhou Key Laboratory for Special Fiber Photonic Devices, South China Normal University, Guangzhou, China, e-mail: wanggh@scnu.edu.cn
Chengkang Liang, Guangdong Provincial Key Laboratory of Nanophotonic Functional Materials and Devices, South China Normal University, Guangzhou, China
Dongxue Liu, Rao Liu, Dongmei Deng, Guangzhou Key Laboratory for Special Fiber Photonic Devices, South China Normal University, Guangzhou, China

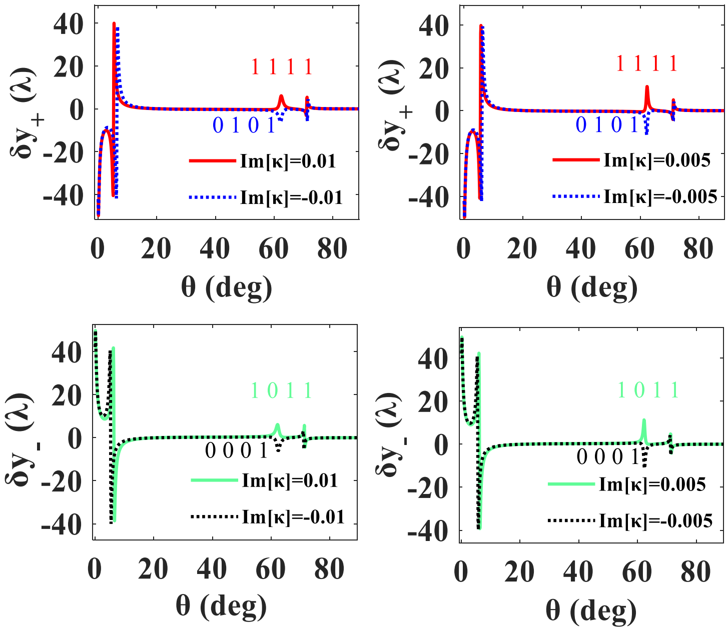

Fig. S1: The coding of "four-digit barcode B" based on TS in the case of Re[κ]=0. Encoding "1 1 1 1" (positive Im[κ]) and "0 1 0 1" (negative Im[κ]) for the LCP light. Realizing "1 0 1 1" (positive Im[κ]) and "0 0 0 1" (negative Im[κ]) for the RCP component.

output codes [0,0], [0,1], [1,0] and [1,1] are completed. So, the binary coding conversion function between input and output codes is perfectly realized. In the barcode encryption function, especially, when the Re[κ] and Im[κ] are both zero, the system will degenerate into a pure PT-symmetrical system, as shown in the third column of the Figure S2. This special point is denoted as "0 0 0 0".

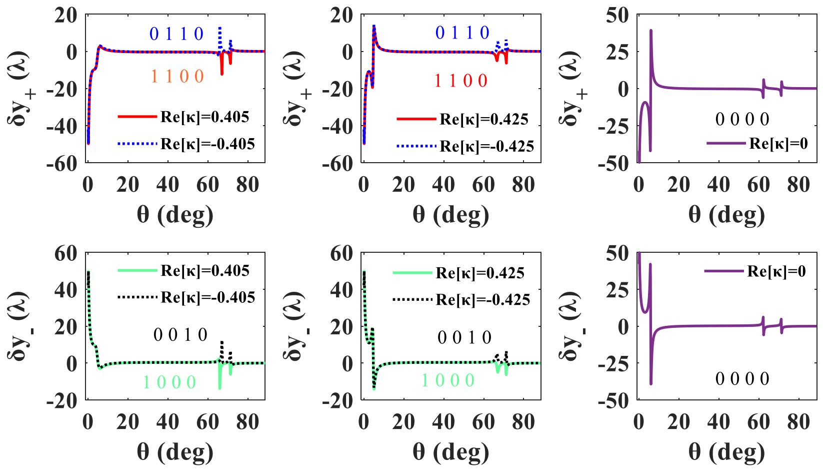

Fig. S2: The implementation of "four-digit barcode B" based on the large TS in the case of Im[κ]=0. Encoding "1 1 0 0" (positive Re[κ]) and "0 1 1 0" (negative Re[κ]) for the LCP component. "1 0 0 0" (positive Re[κ]) and "0 0 1 0" (negative Re[κ]) for the RCP. A specific "0 0 0 0" is defined in the absence of chirality.
